# Identification and Validation of Autophagy-Related Genes in Vitiligo

**DOI:** 10.3390/cells11071116

**Published:** 2022-03-25

**Authors:** Yiwen Yang, Xiuyi Wu, Xiaoli Lu, Chen Wang, Leihong Xiang, Chengfeng Zhang

**Affiliations:** Department of Dermatology, Huashan Hospital, Fudan University, 12 Wulumuqi Zhong Road, Shanghai 200040, China; 18111220037@fudan.edu.cn (Y.Y.); 13301050315@fudan.edu.cn (X.W.); 20211220146@fudan.edu.cn (X.L.); 15301050230@fudan.edu.cn (C.W.)

**Keywords:** autophagy, vitiligo, RNA-sequencing, skin

## Abstract

Vitiligo is a common depigmented disease with unclear pathogenesis. Autophagy is crucial for maintaining cellular homeostasis and has been linked to a variety of autoimmune disorders; however, there have been no reports exploring the involvement of autophagy-related genes (ARGs) in vitiligo using bioinformatics methodologies. In this study, RNA-sequencing technology was used to identify the differentially expressed genes (DEGs) and the Human Autophagy Database (HADb) was overlapped to identify differentially expressed autophagy-related genes (DEARGs) in stable non-segmental vitiligo (NSV). Bioinformatics analyses were conducted with R packages and Ingenuity Pathways Analysis (IPA). DEARGs were further confirmed with qRT-PCR. Critical autophagy markers were detected with Western blotting analysis. We identified a total of 39 DEARGs in vitiligo lesions. DEARGs-enriched canonical pathways, diseases and bio functions, upstream regulators, and networks were discovered. qRT-PCR confirmed the significant increases in FOS and RGS19 in vitiligo lesions. Lower microtubule-associated protein 1 light chain (LC3) II/LC3I ratio and higher sequestosome 1 (SQSTM1, p62) expression were found in vitiligo lesions. In conclusion, this study provided a new insight that autophagy dysregulation appeared in stable vitiligo lesions and might be involved in the etiology of vitiligo by taking part in multiple pathways and bio functions.

## 1. Introduction

Vitiligo is a common depigmented disorder characterized by white patches. Patients with vitiligo normally feel depressed and have trouble socializing. The prevalence of vitiligo in the world population is 0.5–2% [1]. The mechanisms of vitiligo still remain unclear. Previous research studies have demonstrated that intrinsic and extrinsic melanocyte deficiencies, oxidative stress, innate immunological inflammation, T-cell-mediated melanocyte destruction, and loss of melanocyte adhesion may play a role in the pathogenesis of vitiligo [2,3].

Macroautophagy, chaperone-mediated autophagy, and microautophagy are the three types of autophagy. The most well-studied is macroautophagy (also known as “autophagy”). Autophagy is a lysosomal-dependent degradation mechanism that is commonly present in eukaryotic cells. It can regulate the degradation of long-lived proteins and organelles in the cell, and maintain cell homeostasis under starvation, infection, stress, and other conditions. Autophagy has been reported to be associated with multiple autoimmune diseases such as systemic lupus erythematosus (SLE), rheumatoid arthritis (RA), Crohn’s disease, and ankylosing spondylitis (AS) [4]. For instance, the T300A variant of the ATG16lL gene, which plays a key role in autophagosome formation, has been identified as a risk factor for Crohn’s disease [5]. Additionally, autophagy was found to be critical in defending cells from oxidative stress. Our previous work revealed ATG7-dependent autophagy is required for redox homeostasis and the biological processes of melanocytes, such as melanogenesis, proliferation, apoptosis, and senescence [6]. Moreover, it was previously shown that melanocytes of vitiligo patients have dysregulated autophagy and are more sensitive to oxidative stress induced by hydrogen peroxide [7]. Increased ATG7 and ATG8 expression were also found in melanocytes in vitiligo non-lesions [8]. It has been shown that autophagy can also affect the expression of functional molecules related to melanin synthesis. The induction of autophagy enhances the expression of microphthalmia-associated transcription factors and tyrosinases [9]. Ganesan et al. also found a close functional relationship between melanogenesis and autophagy with genome-wide siRNA-based functional genomics [10]. These studies suggest that impaired autophagy might participate in the etiology of vitiligo; however, autophagy-related genes (ARGs) of vitiligo still remain largely unknown. Although exploring ARGs in vitiligo could be beneficial to further improve the pathogenesis of vitiligo and find potential treatments, research studies using bioinformatics to investigate the role of ARGs in vitiligo are still unavailable.

In this study, we performed RNA-sequencing (RNA-seq) using tissue samples from stable vitiligo patients and healthy controls, identified the ARGs, and subsequently explored their upstream regulators, enriched canonical pathways, and biological functions with bioinformatics tools.

## 2. Materials and Methods

### 2.1. Tissue Specimens

Lesion skin tissue specimens were obtained by suction blistering from patients with stable non-segmental vitiligo (NSV). Normal human skin samples were obtained from healthy volunteers subjected to plastic surgery or circumcision. The biopsy specimens were frozen in RNA Later solution (Invitrogen, Waltham, MA, USA) or stored directly at −80 °C for protein extraction. All samples were obtained with patients’ informed consent in Huashan Hospital, affiliated to Fudan University. The study was carried out with ethical approval from the Independent Ethics Committee of Huashan Hospital (Ethical Committee Decision number: 2017-303) and the rules of the Declaration of Helsinki.

### 2.2. RNA Isolation and Library Preparation

Epidermal tissues (*N* = 6 for RNA-seq) were homogenized in a mechanical homogenizer (TissueLyser LT, Qiagen, Venlo, The Netherlands) at 50 Hz for 5 min. Total RNA of patients’ skin samples was extracted using the TRIzol reagent according to the manufacturer’s specification (Invitrogen, Waltham, MA, USA). The purity and quantification of the extracted RNA were evaluated using the NanoDrop 2000 spectrophotometer (Thermo Fisher Scientific, Waltham, MA, USA), and the integrity of the RNA was assessed using the Agilent 2100 Bioanalyzer (Agilent Technologies, Santa Clara, CA, USA). Then, the libraries were constructed using TruSeq Stranded mRNA LT Sample Prep Kit (Illumina, San Diego, CA, USA) according to the manufacturer’s protocols. The transcriptomic sequencing was conducted by Shanghai KR Pharmtech, Inc., Ltd. (Shanghai, China).

### 2.3. ARGs Dataset

A total of 222 ARGs were obtained from the Human Autophagy Database (HADb) (http://www.autophagy.lu/index.html (accessed on 2 December 2021)) (Table S1).

### 2.4. Differentially Expressed Genes (DEGs) Analysis and Ingenuity Pathways Analysis (IPA)

Differential expression analysis was performed using the DESeq R package. *p*-value < 0.05 and absolute log_2_FoldChange (FC) > 0.58 were set as the threshold for significantly differential expression. Principal component analysis (PCA) was performed based on DEGs. The volcano plot and the Venn diagram were drawn using the ggplot2 packages in R. The heatmap was drawn using R packages.

The results of the RNA-seq study were imported into the IPA system (2000–2022 QIAGEN Inc., Düsseldorf, Germany) for core analysis and then overlaid with the global molecular network in the Ingenuity Pathway Knowledge Base (IPKB). Genes’ enriched canonical pathways, diseases, and bio functions, upstream regulators, and gene networks analysis were all investigated using IPA. The IPA uses two scores that address two independent aspects [11]: a *p*-value that measures the overlap of observed and predicted regulated gene sets, and a z-score assessing the match of observed and predicted up/down regulation patterns. In this study, we considered −log(*p*-value) > 1.3 as the threshold for enrichment significance. The z-score is explained as follows: z-score > 0, an active prediction; z-score < 0, an inhibitive prediction; z-score = 0, not predicted as active or inhibitive; z-score = NaN, the data are ineligible for analysis because there are fewer than four analysis-ready molecules in the dataset associated with the pathway. z-score ≥ 2 or ≤−2 are considered significant.

### 2.5. qRT-PCR

The PrimeScript RT Reagent Kit (Takara, Shiga, Japan) was used to generate cDNA (*N* = 8), which was applied as the template for qRT-PCR by ABI QuantStudio 7 Flex (TermoFisher, Waltham, MA, USA) with SYBR Premix ExTaq (Takara, Shiga, Japan) in a 40-cycle PCR. The denaturing, annealing, and extension conditions of each PCR cycle were 95 °C for 30 s, 95 °C for 5 s, and 60 °C for 34 s, respectively. Sequences of primers are listed in Table S2. The 2−ΔΔCq method was used to quantify the expression levels of target genes, with GAPDH as the internal control.

### 2.6. Protein Extraction and Western Blotting

Epidermal tissues (*N* = 5) were homogenized in a mechanical homogenizer (TissueLyser LT, Qiagen, Venlo, The Netherlands) at 50 Hz for 5 min and total protein was extracted from tissue homogenate using the tissue lysis buffer (Beyotime, Shanghai, China). The BCA Protein Assay Kit (Beyotime, Shanghai, China) was used to determine the protein concentration. The proteins were separated on a 12% Bis-Tris protein gel (GenScript, Piscataway, NJ, USA) and electro-transferred onto NC membranes (Millipore, Burlington, MA, USA), then blocked with QuickBlock blocking buffer (Beyotime, Shanghai, China) and incubated overnight with primary antibodies at 4 °C. After three washes with TBS-T (Biosharp, Hefei, China; Solarbio, Beijing, China), the membranes were incubated with horseradish peroxidase (HRP)-conjugated secondary antibodies at room temperature for 1 h. The following antibodies were used: anti-GAPDH (#5174, CST, Danvers, MA, USA); anti-microtubule-associated protein 1 light chain (LC3) A/B (#12741, CST, Danvers, MA, USA); anti- sequestosome 1 (SQSTM1, p62) (#ab109012, Abcam, Cambridge, UK); and anti-rabbit IgG, HRP-linked Antibody (#7074, CST, Danvers, MA, USA). Immunoreactive bands were detected by ultra-sensitive ECL chemiluminescent substrate (Biosharp, Hefei, China) and visualized on a Tanon 5200 scanner (Tanon, Shanghai, China) with GelCap software (Tanon, Shanghai, China).

### 2.7. Statistical Analysis

The student’s *t*-test was used for analyzing qRT-PCR and western blotting results from two groups. *p* < 0.05 was considered statistically significant. Statistical analyses were performed using GraphPad Prism 8 (GraphPad Software Inc., San Diego, CA, USA).

## 3. Results

### 3.1. Identification of DEGs in Vitiligo Lesions

**Figure 1:**
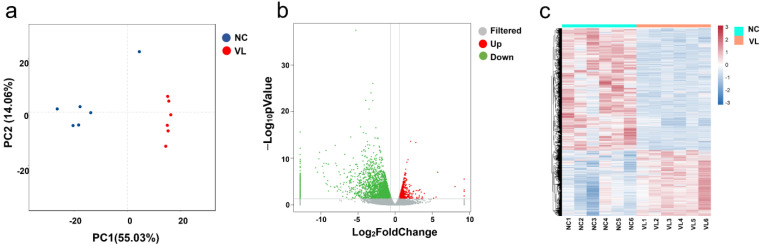
Differentially expressed genes (DEGs) in vitiligo lesions compared with normal controls. (**a**) Principal component analysis (PCA) of RNA-seq dataset. (**b**) Volcano plot displaying differentially expressed genes between vitiligo lesions and healthy controls under the cut-off: *p* value < 0.05 and absolute log_2_FoldChange > 0.58. Green dots: significantly downregulated genes; red dots: significantly upregulated genes; grey dots: filtered genes. (**c**) Heatmap of DEGs. NC: normal control; VL: vitiligo lesion.

Principal component analysis (PCA) showed that the samples were clearly separated by groups and there was a large distance between normal controls and vitiligo samples (Figure 1a). We eventually obtained a total of 4030 significant DEGs, of which 1408 were upregulated and 2622 were downregulated, using the criteria of absolute log_2_FC > 0.58 and *p*-value < 0.05 (Figure 1b), whereas the expression pattern of DEGs in heathy samples and vitiligo lesions is shown in the hierarchical heatmap (Figure 1c). Among them, a lot of genes have been described to be involved in vitiligo, such as CXCL9 [12], CCR6 [13], and IL-33 [14]. The top five downregulated genes were KRT25, LRRC15, KRT85, KRT71, and SEZ6L. The top five upregulated genes were CPA3, GCSAML, LBX1, PPBP, and TPSAB1. ARGs were not among the most regulated genes. We further explored DEARGs in vitiligo lesions with HADb.

### 3.2. Identification of Differentially Expressed Autophagy-Related Genes (DEARGs) in Vitiligo Lesions

**Figure 2:**
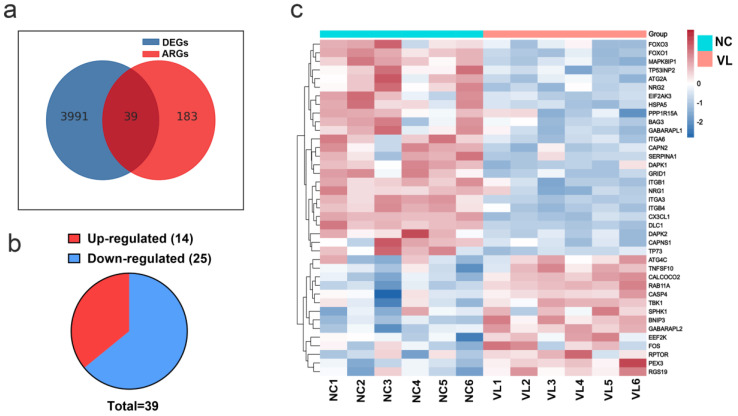
Differentially expressed autophagy-related genes (DEARGs)’ expression in vitiligo lesions. (**a**) Genes overlapped by the differentially expressed genes (DEGs) in vitiligo lesions and the autophagy-related genes (ARGs). (**b**) The pie graph of upregulated and downregulated DEARGs. (**c**) Heatmap of DEARGs. NC: normal control; VL: vitiligo lesion.

A total of 222 ARGs were downloaded from the HADb. The Venn figure (Figure 2a) shows there were 39 DEARGs in vitiligo lesions, including 14 upregulated genes and 25 downregulated genes (see Figure 2b, Table 1 for details). The top five upregulated genes were BNIP3, TNFSF10, FOS, PEX3, and RGS19, and the top five downregulated genes were DAPK1, SERPINA1, GRID1, CX3CL1, and DLC1. Furthermore, a heatmap was created to show the expression pattern of 39 DEARGs in controls and vitiligo samples (Figure 2c).

### 3.3. Canonical Pathway Analysis of DEARGs

**Figure 3:**
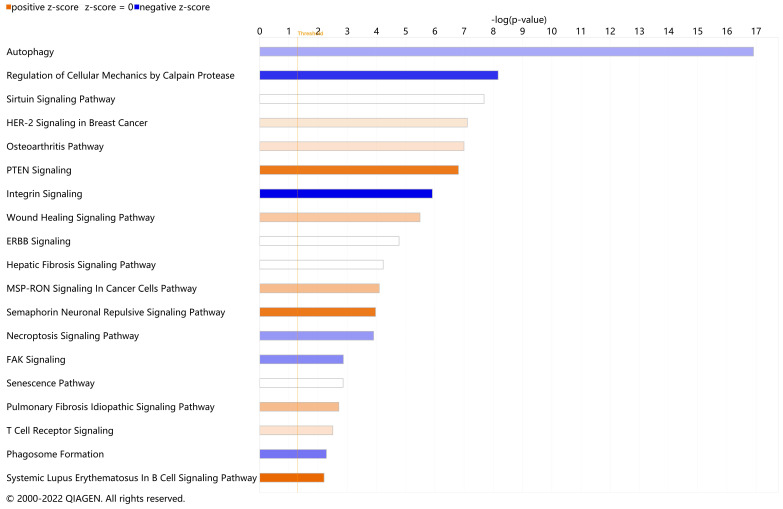
Enriched canonical pathways of differentially expressed autophagy-related genes (DEARGs) in vitiligo. Blue band: the negative prediction of the pathway; orange band: the active prediction of the pathway; white band: the pathways that could not be predicted to be activated or suppressed. The filter was absolute z-score ≥ 0.0. The threshold line was drawn at −log(*p*-value) = 1.3.

To elucidate the DEARGs-enriched pathways, we uploaded these DEARGs to IPA software for core analysis and then performed the canonical pathway analysis. A total of 104 enriched canonical pathways were discovered under the threshold of −log(*p*-value) > 1.3. The full canonical pathways are shown in Table S3. A total of 19 enriched canonical pathways were identified under the criteria of −log(*p*-value) > 1.3 and absolute z-score ≥ 0.0 (Figure 3). Among them, six pathways were found to be inhibited (z-score < 0.0), nine pathways were found to be activated (z-score > 0.0), and four pathways showed no inhibition or activation prediction (z-score = 0). The top five canonical pathways were ‘Autophagy’ (−log(*p*-value) = 16.900, z-score = −0.832), ‘Regulation of Cellular Mechanics by Calpain Protease’ (−log(*p*-value) = 8.170, z-score = −2.000), the ‘Sirtuin Signaling Pathway’ (−log(*p*-value) = 7.680, z-score = 0.000), ‘HER-2 Signaling in Breast Cancer’ (−log(*p*-value) = 7.110, z-score = 0.378), and the ‘Osteoarthritis Pathway’ (−log(*p*-value) = 7.000, z-score = 0.447). The highest-ranked pathway, ‘Autophagy’, confirmed the reliability of selecting our DEARGs from the HADb. Taking an absolute z-score value greater than 2.0 as the significant prediction threshold, it was subsequently found that ‘PTEN Signaling’ (z-score = 2.000), the ‘Semaphorin Neuronal Repulsive Signaling Pathway’ (z-score = 2.000), and the ‘Systemic Lupus Erythematosus In B Cell Signaling Pathway’ (z-score = 2.236) were significantly activated; ‘Regulation of Cellular Mechanics by Calpain Protease’ (z-score = −2.000) and ‘Integrin Signaling’ (z-score = −2.449) were significantly inhibited.

### 3.4. Diseases and Bio Function Analysis of DEARGs

**Figure 4:**
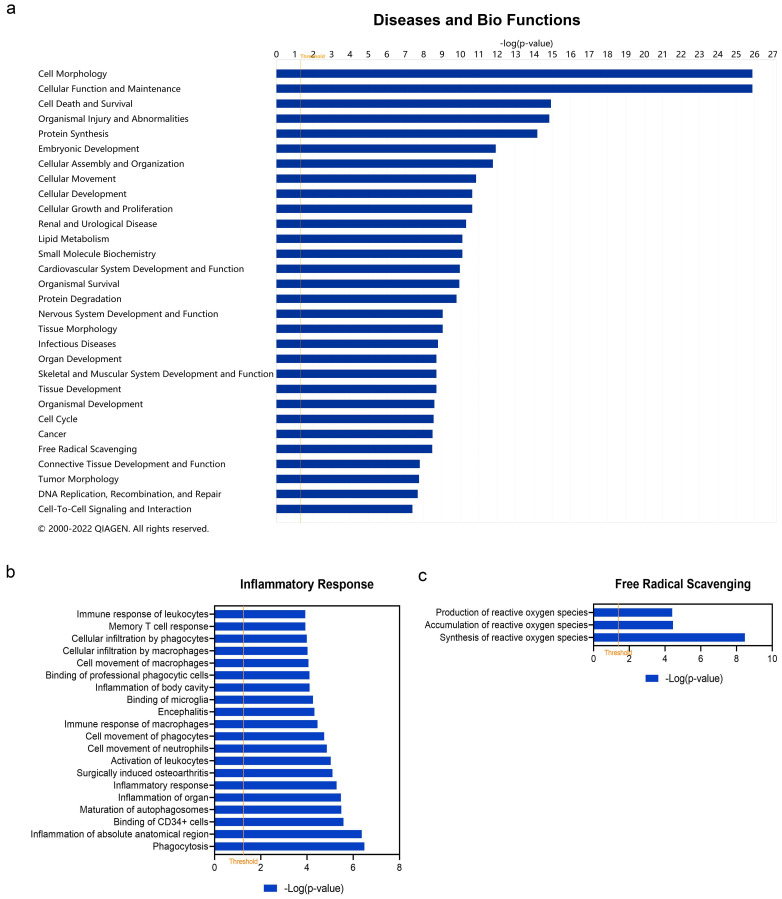
Enriched diseases and bio functions of differentially expressed autophagy-related genes (DEARGs) in vitiligo. (**a**) Top 30 enriched diseases and bio functions of DEARGs. (**b**) Inflammatory response of DEARGs. (**c**) Free radical scavenging of DEARGs. The threshold line was drawn at −log(*p*-value) = 1.3.

In addition to canonical pathways, DEARGs were also categorized to related diseases and functions. The top 30 diseases and Bio Functions ranked by −log(*p*-value) are shown in Figure 4a. The full results are shown in Table S4. DEARGs were found to be mostly enriched in ‘Cell Morphology’, ‘Cellular Function and Maintenance’, ‘Cell Death and Survival’, ‘Organismal Injury and Abnormalities’, ‘Protein Synthesis’, etc. Since vitiligo is considered as one kind of inflammatory disease, the role of DEARGs on ‘Inflammatory Response’ was explored in detail (Figure 4b, see details in Table S5). It was found that these DEARGs were involved in ‘Inflammation of Organ’ (−log(*p*-value) = 5.4698, z-score = 2.155), ‘Inflammatory response’ (−log (*p*-value) = 5.281, z-score = −1.254), ‘Activation of leukocytes’ (−log(*p*-value) = 5.029, z-score = −0.035), ‘Memory T cell response’ (−log(*p*-value) = 3.932, z-score = null), ‘Immune response of leukocytes’ (−log(*p*-value) = 3.928, z-score = −0.956), etc. Since oxidative stress is considered the triggering factor of vitiligo, we also studied the category of ‘Free Radical Scavenging’ in detail (Figure 4c, see details in Table S6). The result showed these DEARGs played a role in ‘Synthesis of reactive oxygen species’ (−log(*p*-value) = 8.469, z-score = −1.062), ‘Accumulation of reactive oxygen species’ (−log(*p*-value) = 4.442, z-score = null), and ‘Production of reactive oxygen species’ (−log(*p*-value) = 4.400, z-score = −1.309).

### 3.5. Upstream Regulator Analysis of DEARGs

**Figure 5:**
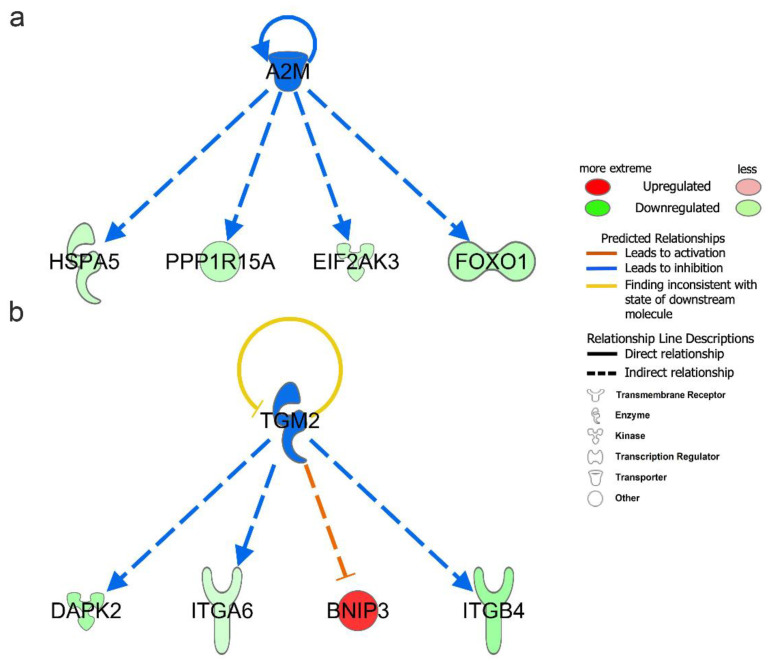
Upstream regulator analysis of differentially expressed autophagy-related genes (DEARGs) in vitiligo. (**a**) Downstream targets of A2M among the DEARGs. (**b**) Downstream targets of TGM2 among the DEARGs.

Upstream regulators include genes, RNAs, proteins, drugs, and chemicals. To better understand the regulatory mechanism of DEARGs changes, we performed upstream regulator analysis with the filter of molecule type (genes, RNAs, and proteins included) in IPA. A total of 1026 upstream regulators were found under the criteria of *p*-value < 0.05. The upstream regulators are shown in Table 2 under the criteria of *p*-value < 0.05 and absolute z-score > 1.0. The full upstream regulators are shown in Table S7. Among them, A2M (−log(*p*-value) = 5.963, z-score = −2.000), SYVN1 (−log(*p*-value) = 3.943, z-score = −2.000), and TGM2 (−log(*p*-value) = 3.188, z-score = −2.000) were predicted to be significant inhibitors. Additionally, SIRT1 (−log(*p*-value) = 5.000, z-score = 2.236) was predicted to be the significant activator. Moreover, A2M and TGM2 upstream regulators were also found to be differentially expressed in vitiligo samples. In our DEARGs, the downstream targets of A2M were EIF2AK3, FOXO1, HSPA5, and PPP1R15A (Figure 5a); the corresponding targets of TGM2 were BNIP3, DAPK2, ITGA6, and ITGB4 (Figure 5b).

### 3.6. Molecular Network Analysis of DEARGs

**Figure 6:**
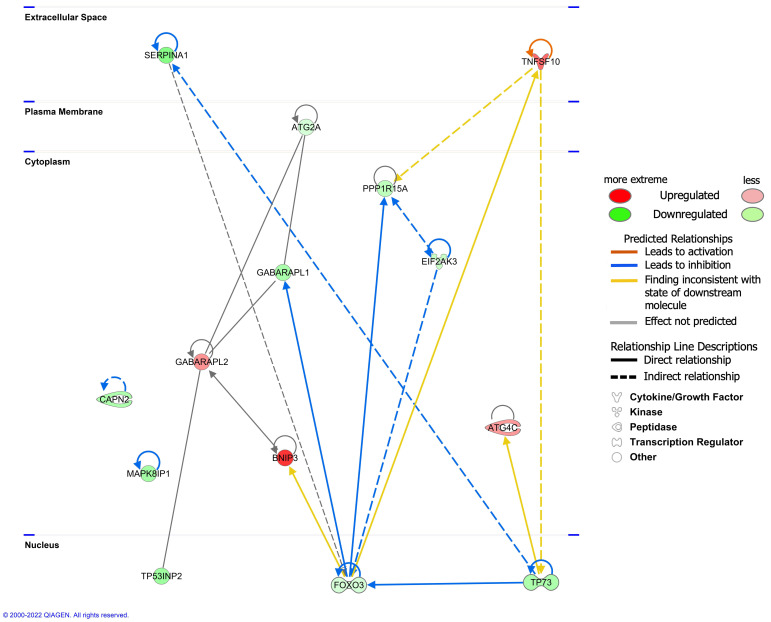
The interaction of 14 differentially expressed autophagy-related genes (DEARGs) involved in the ‘Cell Morphology, Cellular Function and Maintenance, Cell Death and Survival’ network.

The interaction network analysis revealed the interactions between molecules in the dataset. All networks were then sorted using the score values. The networks were supplemented (Table S8). The highest-ranked network (score 33) was found to mainly affect ‘Cell Morphology, Cellular Function and Maintenance, Cell Death and Survival’, involving 14 molecules in our DEARG dataset: ATG2A, ATG4C, BNIP3, CAPN2, EIF2AK3, FOXO3, GABARAPL1, GABARAPL2, MAPK8IP1, PPP1R15A, SERPINA1, TNFSF10, TP53INP2, and TP73. The interaction of these 14 DEARGs is shown in Figure 6.

### 3.7. Validation of DEARGs’ Expression in Clinical Samples

**Figure 7:**
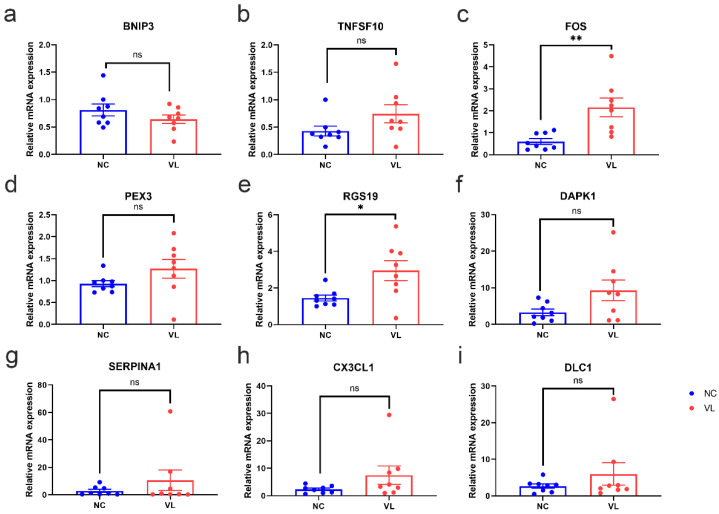
The mRNA expression levels of top regulated differentially expressed autophagy-related genes (DEARGs) in vitiligo lesions and healthy individuals. The epidermis of healthy controls and vitiligo lesions were processed for RNA isolation and gene expression of (**a**) BNIP3, (**b**) TNFSF10, (**c**) FOS, (**d**) PEX3, (**e**) RGS19, (**f**) DAPK1, (**g**) SERPINA1, (**h**) CX3CL1 (**i**) DLC1 were evaluated by qRT-PCR. Expression was calculated using the 2^−ΔΔCt^ method. Data were summarized from eight biological replicates and values are shown as the mean ± SEM. NC: normal control; VL: vitiligo lesion. *: *p* < 0.05; **: *p* < 0.01; ns: non-significant.

To validate the reliability of our RNA-seq dataset, the expression levels of top regulated DEARGs with base mean values higher than 10 were further identified by qRT-PCR in our clinical samples. Consistent with the results of our RNA-seq data, the expression levels of FOS (1.554 ± 0.4429, *p* = 0.0035) and RGS19 (1.497 ± 0.5682, *p* = 0.0196) were significantly higher in vitiligo lesions than in normal samples, while the expression levels of BNIP3 (−0.1705 ± 0.1336, *p* = 0.2226), TNFSF10 (0.3146 ± 0.1865, *p* = 0.1137), PEX3 (0.3394 ± 0.2225, *p* = 0.1495), DAPK1 (6.013 ± 2.973, *p* = 0.0627), SERPINA1 (7.702 ± 7.544, *p* = 0.3246), CX3CL1 (5.194 ± 3.359, *p* = 0.1443), and DLC1 (3.315 ± 3.131, *p* = 0.3077) showed no significant difference between two groups (Figure 7).

### 3.8. Autophagy Expression in Vitiligo Lesions

**Figure 8:**
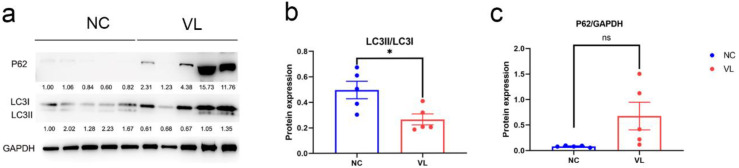
The protein expression levels of LC3 and p62 in vitiligo lesions and healthy controls. (**a**) Western blotting image of LC3I, LC3II and p62 expression in vitiligo samples and healthy controls. The indicated protein expression by densitometry analysis relative to the level in the first lane is represented by the numerical values displayed below the bands. (**b**) Semi-quantification of the relative ratio of LC3II/LC3I; (**c**) Semi-quantification of the relative ratio of p62/GAPDH. Data are shown from 5 biological replicates and values are shown as the mean ± SEM. NC: normal control; VL: vitiligo lesion. *: *p* < 0.05; ns: non-significant.

To evaluate the autophagy status in vitiligo patients, we compared LC3 expression and p62 expression in vitiligo samples and healthy individuals, which are two important markers of autophagy. Our results showed a significantly decreased ratio of LC3II/LC3 (−0.2316 ± 0.0808, *p* = 0.0210) and elevated level of p62 (0.5944 ± 0.2713, *p* = 0.0599) in vitiligo lesions (Figure 8).

## 4. Discussion

In the present study, we performed RNA-sequencing using vitiligo samples and healthy controls and then conducted bioinformatic analysis with IPA. The HADb is the first human autophagy-dedicated database, which is a public repository containing information about the human genes described so far as involved in autophagy. After overlapping it with DEGs in vitiligo samples, we identified 39 DEARGs (BNIP3, TNFSF10, FOS, PEX3, RGS19, GABARAPL2, EEF2K, CASP4, CALCOCO2, ATG4C, TBK1, RAB11A, RPTOR, SPHK1, ATG2A, FOXO3, ITGA6, CAPNS1, EIF2AK3, HSPA5, ITGB1, BAG3, PPP1R15A, FOXO1, NRG2, ITGA3, CAPN2, TP73, NRG1, GABARAPL1, DAPK2, MAPK8IP1, ITGB4, TP53INP2, DAPK1, SERPINA1, GRID1, CX3CL1, and DLC1) in vitiligo lesions under the criteria of absolute log_2_FC > 0.58 and *p*-value < 0.05.

IPA results provided some new insights into the role of autophagy in vitiligo pathogenesis. Canonical pathway analysis showed the highest enriched pathway was ‘Autophagy’ (ATG2A, BNIP3, DAPK1, DAPK2, EIF2AK3, FOS, FOXO1, FOXO3, GABARAPL1, GABARAPL2, RGS19, RPTOR, and TBK1) and ‘Autophagy’ was predicted to be inhibited (z-score = −0.832) (Figure S1), which was consistent with our subsequent finding showing the lower ratio of LC3II/LC3I and higher p62 expression in vitiligo lesions. Under canonical pathway analysis, ‘PTEN Signaling’ (FOXO1, FOXO3, ITGA3, ITGA6, ITGB1, and ITGB4), the ‘Semaphorin Neuronal Repulsive Signaling Pathway’ (ITGA3, ITGA6, ITGB1, and ITGB4), and the ‘Systemic Lupus Erythematosus In B Cell Signaling Pathway’ (FOS, FOXO1, FOXO3, TBK1, and TNFSF10) were predicted to be significantly activated. PTEN is a tumor-suppressor protein that modulates signaling pathways involved in cell growth, migration, and apoptosis by acting as a dual specificity protein phosphatase and an inositol phospholipid phosphatase. The activation prediction of PTEN signaling is consistent with another study claiming high PTEN expression in vitiligo patients. PTEN overexpression could lead to oxidative stress-induced melanocyte apoptosis and eventually the initiation of vitiligo. That study also claimed mesenchymal stem cells enhanced the cell proliferation of melanocytes and rescued melanocytes from oxidative stress via downregulating PTEN expression. PTEN inhibitors might be one therapeutic strategy for vitiligo. Our study revealed that autophagy-related genes were also involved in PTEN signaling regulation, which suggested that targeting autophagy might be beneficial for vitiligo patients by regulating PTEN signaling [15]. The activation prediction of PTEN signaling was mainly due to the downregulation of FOXOs and integrins. FOXO transcription factors could combine signals from food restriction and stress stimuli in order to coordinate gene programs involved in cellular metabolism and oxidative stress resistance [16]. One study indicated that rs4946936 of the FOXO3A gene might be associated with susceptibility to vitiligo, especially active vitiligo, and FOXO3A levels were decreased in vitiligo patients compared with normal controls [17]. Another study reported the downregulation of FOXO1 in vitiligo lesional skin [18]. Integrins are proteins that link the cell cytoskeleton to the extracellular matrix (ECM) mechanically and biochemically. Our result revealed a lower expression pattern of ITGA3, ITGA6, ITGB1, and ITGB4 in vitiligo lesions, which is consistent with a former study by Adriane Reichert Faria demonstrating a significant reduction in integrin expression in vitiligo skin and claiming the contribution of integrin deficiency in the adhesion impairment in vitiligo [19]. One study found that Integrin Signaling was the top pathway upregulated in the narrow-band UVB (NBUVB)-treated bulge melanocytes [20] and integrins were found to have promigratory effects on melanocytes [21]. Our study showed an inhibitory effect of autophagy-related genes in vitiligo lesions on Integrin Signaling, which suggested that targeting autophagy might be beneficial for vitiligo by regulating Integrin Signaling. Semaphorins have been implicated in cell migration, tumor growth, and immune response. The activation prediction of the ‘Semaphorin Neuronal Repulsive Signaling Pathway’ was also mainly due to the alterations of integrin proteins. Since both systemic lupus erythematosus and vitiligo are autoimmune diseases, the predicted activation of the ‘Systemic Lupus Erythematosus In B Cell Signaling Pathway’ suggested that autophagy might play a role in the autoimmune disease, which has been confirmed by other studies [22,23]. Besides FOS, FOXO1, and FOXO3, upregulated TBK1 and TNFSF10 contributed to the activation prediction of ‘Systemic Lupus Erythematosus In B Cell Signaling’. TBK1 is mainly known for its role in innate immunity antiviral responses. Indeed, viral factor has been implicated in the etiopathogenesis of vitiligo. Phosphorylation of TBK1 could be found under the intra- or extracellular poly(I:C) stimulation in melanocytes [24]. TNFSF10 is a cytokine that belongs to the tumor necrosis factor (TNF) ligand family. A positive correlation between TNFSF10 and macrophage abundance has actually been found in vitiligo [25].

There were two enriched significantly inhibited pathways: ‘Regulation of Cellular Mechanics by Calpain Protease’ (CAPN2, CAPNS1, ITGA3, ITGA6, ITGB1, and ITGB4) and ‘Integrin Signaling’ (CAPN2, CAPNS1, ITGA3, ITGA6, ITGB1, and ITGB4). In addition to integrin proteins, the alterations of CAPNs also contributed to the inhibition prediction of these two signaling pathways. CAPN codes for calpains, a family of proteases strongly associated with oxidative stress. CAPN3 overexpression was found in the skin of vitiligo vulgaris Mexican patients [26]. However, the expression of CAPN2 and CAPNS1 in vitiligo needs to be further explored. Taken together, our findings showed that autophagy might play a role in the etiology of vitiligo by regulating multiple signaling pathways.

The potential diseases and bio function analysis showed DEARGs in vitiligo were enriched in ‘Cell Morphology’, ‘Cellular Function and Maintenance’, ‘Cell Death and Survival’, ‘Organismal Injury and Abnormalities’, etc. Molecular network analysis also scored the ‘Cell Morphology, Cellular Function and Maintenance, Cell Death and Survival’ as the highest-ranked molecular network of DEARGs in vitiligo lesions, which further confirmed the crucial role of autophagy in maintaining cellular homeostasis and normal physiological functions. This result is consistent with our previous finding that autophagy is critical for redox homeostasis and the biological functions of human melanocytes, such as melanogenesis, proliferation, apoptosis, and senescence, especially under oxidative stress [6], and that autophagy-deficient murine melanocytes developed premature senescence and accumulated products of oxidative damage [27]. Actually, autophagy was also shown to have a role in a variety of physiological processes in keratinocytes, including apoptosis, differentiation, inflammation, and melanin metabolism [28].

Autoimmune and oxidative stress have been considered as the critical factors in the onset and progression of vitiligo. Therefore, we studied the ‘Inflammatory Response’ and ‘Free Radical Scavenging’ in detail. Our results showed these DEARGs significantly activate the ‘Inflammation of Organ’ and showed inhibitory effects in ‘Inflammatory Response’, ‘Activation of leukocytes’, and ‘Immune response of leukocytes’. ‘Memory T cell response’ was involved, but its z-score was null, which might have been due to the fewer analysis-ready molecules in the dataset associated with this function. Other studies also demonstrated the involvement of autophagy in inflammatory diseases such as lupus erythematosus [22], and inflammatory bowel disease [23]. As far as ‘Free Radical Scavenging’ is concerned, these DEARGs showed an inhibitory effect in the synthesis and production of reactive oxygen species, which has also been claimed in other studies [29,30].

We further explored the upper regulators of DEARGs in vitiligo lesions. A2M and TGM2 were predicted to be the inhibitory upstream regulators of the DEARGs in vitiligo. Moreover, A2M and TGM2 were shown to be downregulated in vitiligo lesions from our RNA-seq dataset, which more favorably indicates that their decreased expression might subsequently cause the expression alterations of the downstream DEARGs. A2M and related proteins in the plasma and tissues of vertebrates serve as humoral defense barriers against pathogens by binding host or foreign peptides and particles [31]. TGM2 is an enzyme that catalyzes the crosslinking of proteins by epsilon-gamma glutamyl lysine isopeptide bonds. There were studies showing that A2M played an important role in the function of many immune cells [32], and TGM2 was involved in several autoimmune diseases such as celiac disease [33], inflammatory bowel disease, osteoarthritis, and idiopathic inflammatory myopathies [34]. However, little is known about the function of A2M and TGM2 in vitiligo, which needs to be explored in the future.

After experimental verification for several markedly altered candidates, FOS and RGS19 were confirmed to be significantly high in vitiligo lesions, which was in line with the results from our RNA-seq dataset. FOS is one of the AP-1 transcription factor subunits. AP-1 regulates several cellular processes, including differentiation, proliferation, and apoptosis [35]. FOS was found to be highly expressed in the PIG1 melanocyte cell line and primary human epidermal melanocytes under oxidative stress. The upregulated FOS might contribute to melanocyte apoptosis. Targeting FOS might be helpful for melanocyte survival under stress, which needs to be confirmed in the future [36]. Another study explored the relationship between immune cell infiltration and gene expression in vitiligo by combining bioinformatics methods and expression analysis techniques, and claimed that FOS expression was positively correlated with macrophage abundance [25], which has been demonstrated in vitiligo lesions [37,38]. Indeed, macrophages are involved in the clearing of apoptotic melanocytes from the skin in vitiligo patients [39].

The protein encoded by RGS19 is a guanosine triphosphatase-activating protein that functions to down-regulate Galpha i/Galpha q-linked signaling. Little is known about RGS19 in vitiligo. RGS19-deficient animals had abnormal antibody responses, B cell trafficking abnormalities, and a larger B cell compartment in the spleen [40], which suggested an essential role of RGS19 in the immune response. However, the role of RGS19 in the immune response of vitiligo still needs further study.

Finally, we detected expression of LC3 and p62 in vitiligo lesions, which are the crucial biological markers to identify autophagy in mammalian systems. LC3-1 in the cytosol is conjugated to phosphatidylethanolamine during autophagy to create LC3-II, which is then integrated into the autophagosomal membrane. For the autophagic degradation of substrates, p62 works as a cargo receptor [41]. Our study revealed a lower LC3II/LC3I ratio and higher p62 expression in vitiligo lesions, which suggested that autophagy was inhibited in vitiligo lesions. Consistent with our results, Rehab M Naguib et al. found the LC3 expression in vitiligo lesions was significantly lower as compared with normal controls [42]. Dysregulation of autophagy might cause the accumulation of reactive oxygen species in situ and subsequently trigger the onset of vitiligo. Emanuela Bastonini et al. found ATG7 was highly expressed in non-lesional vitiligo melanocytes compared with healthy controls and demonstrated a protective role of autophagy for vitiligo [8]. Our previous study indicated that ATG7-dependent autophagy was indispensable for redox homeostasis and the biological functions of melanocytes. ATG7-deficient melanocytes produce less melanin and less melanogenesis-associated proteins such as tyrosinase (TYR), TYR-related protein 2 (TRP2), and microphthalmia-associated transcription factor (MITF). ATG7 deficiency could inhibit melanocyte proliferation and facilitate oxidative stress-driven apoptosis [6]. Another study found higher expression of ATG5 in the lesional and perilesional skin of both active and stable vitiligo patients and it claimed that higher expressed ATG5 might induce apoptosis in the perilesional skin of active vitiligo patients by autophagy-independent functions [43], while Haiyan Yu et al. found ATG5 and ATG12, key components of autophagy formation, were significantly reduced in melanocytes of vitiligo patients under oxidative stress [44]. The studies of autophagy levels in vitiligo seem to not always be consistent with each other, which might be due to the small sample size, different approaches to tissue extraction, and different research objects involved in different studies, with several studies exploring autophagy expression in the whole skin and some studies exploring the role of autophagy in one specific cell type. In this study, we explored autophagy-related genes and autophagy expression in the epidermis. Therefore, our results are more indicative of the autophagy expression in keratinocytes, which are the main cellular component of the epidermis, in vitiligo patients. The decrease in autophagy level at stable vitiligo epidermis might be due to the loss of melanocytes in the epidermis, congenital autophagy dysfunction of keratinocytes, and subsequent autophagy exhaustion resulting from excessive increase during the progression of the disease, which needs to be further explored. Other research studies also claimed autophagy was involved in the pathogenesis of vitiligo. For instance, the ultraviolet radiation resistance-associated gene (UVRAG), an autophagy-related gene, has the ability to activate the PI3KC3 complex, encouraging autophagy. A Korean study found that the UVRAG polymorphism may have a role in higher susceptibility to NSV in the Korean population, implying a link between autophagy and NSV susceptibility [45].

There are several limitations in our research. Firstly, we only explored the potential DEARGs in stable vitiligo lesions. The role of autophagy in the progression of vitiligo remains to be discovered in the future. Secondly, due to the small sample size of this study and the different methods used to collect the samples, interpretation of the study results needs to be conservative. Finally, the specific pathophysiological mechanisms of DEARGs regulating the initiation and progression of vitiligo need to be further studied and confirmed.

## 5. Conclusions

In summary, we identified 39 DEARGs in vitiligo lesions and conducted several bioinformatics analyses with IPA. FOS and RGS19 were confirmed to have significant increases in vitiligo patients. Moreover, we found the autophagy was inhibited in stable vitiligo lesions. Collectively, the above results indicate that autophagy dysregulation might take part in the pathogenesis of vitiligo. Future studies are required to further explore the detailed function of autophagy in the onset and progression of vitiligo.

## Figures and Tables

**Table 1:** The 39 differentially expressed autophagy-related genes (DEARGs) in vitiligo lesions compared to healthy samples.

Gene Symbol	FoldChange	log_2_FoldChange	*p*-Value	q-Value	Regulation
BNIP3	2.5595679	1.3559003	3.72 × 10^−7^	2.77 × 10^−5^	Up
TNFSF10	1.9768091	0.9831735	0.0002049	0.0041653	Up
FOS	1.927316	0.9465932	0.0411468	0.1800061	Up
PEX3	1.7269818	0.7882529	0.0024115	0.0264225	Up
RGS19	1.6882659	0.7555421	0.0109968	0.0752503	Up
GABARAPL2	1.6506423	0.7230275	0.0049887	0.0436477	Up
EEF2K	1.6256503	0.7010169	0.0066433	0.05304	Up
CASP4	1.6253708	0.7007689	0.0074996	0.0576006	Up
CALCOCO2	1.6038901	0.6815753	0.007666	0.0582566	Up
ATG4C	1.5917797	0.6706407	0.0318542	0.1519605	Up
TBK1	1.5631494	0.6444557	0.0174337	0.1019376	Up
RAB11A	1.5397015	0.6226507	0.0129811	0.0843511	Up
RPTOR	1.524999	0.6088083	0.0185034	0.1058851	Up
SPHK1	1.5135892	0.5979737	0.0352007	0.1624093	Up
ATG2A	0.6509159	−0.619457	0.0220707	0.1191685	Down
FOXO3	0.6484999	−0.624822	0.0372126	0.1685346	Down
ITGA6	0.6116547	−0.709211	0.004182	0.0385211	Down
CAPNS1	0.5507554	−0.860516	0.0368733	0.1673774	Down
EIF2AK3	0.5495273	−0.863737	0.0190598	0.1082081	Down
HSPA5	0.545728	−0.873746	0.0036939	0.0351965	Down
ITGB1	0.5452697	−0.874958	0.0005796	0.0093808	Down
BAG3	0.5318967	−0.910782	0.0306816	0.1481352	Down
PPP1R15A	0.493106	−1.02003	0.0158813	0.0965559	Down
FOXO1	0.4646661	−1.105734	2.24 × 10^−5^	0.0007508	Down
NRG2	0.4609473	−1.117326	0.0032613	0.032505	Down
ITGA3	0.4119834	−1.279342	7.33 × 10^−7^	4.81 × 10^−5^	Down
CAPN2	0.410229	−1.285498	0.0002022	0.0041223	Down
TP73	0.4091502	−1.289298	0.0020614	0.0235629	Down
NRG1	0.4035998	−1.309002	0.0002356	0.0046652	Down
GABARAPL1	0.392561	−1.349011	0.0102162	0.0713961	Down
DAPK2	0.3887637	−1.363035	0.0001956	0.0040326	Down
MAPK8IP1	0.3750976	−1.414662	0.0001184	0.0027454	Down
ITGB4	0.3544888	−1.496188	9.37 × 10^−8^	9.50 × 10^−6^	Down
TP53INP2	0.3456572	−1.532586	0.0128949	0.0840035	Down
DAPK1	0.2971078	−1.750942	6.87 × 10^−8^	7.48 × 10^−6^	Down
SERPINA1	0.2734992	−1.870392	0.0052844	0.0453088	Down
GRID1	0.1295931	−2.94794	0.0035153	0.0340916	Down
CX3CL1	0.1253904	−2.995501	4.33 × 10^−23^	1.56 × 10^−19^	Down
DLC1	0.0883675	−3.500341	2.79 × 10^−21^	8.37 × 10^−18^	Down

**Table 2:** Upstream regulator analysis of differentially expressed autophagy-related genes (DEARGs) in vitiligo lesions.

Upstream Regulator	Molecule Type	Predicted Activation State	Activation z-Score	*p*-Value of Overlap	Target Molecules in Dataset
ERK1/2	group		−1.708	5.14 × 10^−8^	CAPN2, DAPK1, EIF2AK3, FOS, FOXO1, HSPA5, ITGA3, MAPK8IP1
TP63	transcription regulator		−1.471	1.13 × 10^−7^	ATG4C, FOS, FOXO3, ITGA3, ITGA6, ITGB1, ITGB4, TNFSF10, TP73
TGFB1	growth factor		−1.203	4.52 × 10^−7^	CALCOCO2, CAPNS1, CASP4, CX3CL1, DAPK1, FOS, FOXO1, FOXO3, HSPA5, ITGA3
PGR	ligand-dependent nuclear receptor		−1.633	8.11 × 10^−7^	CAPN2, FOS, FOXO1, ITGA6, ITGB1, ITGB4, SERPINA1
NFKBIA	transcription regulator		1.768	1.02 × 10^−6^	BNIP3, CASP4, CX3CL1, FOS, HSPA5, ITGA3, ITGB1, TNFSF10
A2M	transporter	Inhibited	−2	1.09 × 10^−6^	EIF2AK3, FOXO1, HSPA5, PPP1R15A
ATF3	transcription regulator		−1.565	3.92 × 10^−6^	CX3CL1, HSPA5, PPP1R15A, RAB11A, TP73
TP73	transcription regulator		−1.689	6.06 × 10^−6^	ATG4C, CASP4, CX3CL1, FOXO3, ITGB4, SERPINA1, TP73
SIRT1	transcription regulator	Activated	2.236	0.00001	BNIP3, EIF2AK3, FOXO1, FOXO3, GABARAPL1, HSPA5, TP73
AKT1	kinase		1.181	0.000012	FOS, FOXO1, ITGB1, RAB11A, TNFSF10, TP73
HRAS	enzyme		−1.342	2.13 × 10^−5^	BNIP3, CAPN2, FOS, FOXO1, ITGA6, ITGB1, ITGB4, TP73
TCF4	transcription regulator		−1.633	3.35 × 10^−5^	BNIP3, CASP4, FOXO1, ITGA3, TBK1, TNFSF10
Pkc(s)	group		1.215	3.82 × 10^−5^	FOS, HSPA5, ITGB1, PPP1R15A, TP73
CREB1	transcription regulator		−1.151	5.28 × 10^−5^	BAG3, BNIP3, FOS, FOXO3, HSPA5, PPP1R15A, TP53INP2
FSH	complex		1.969	6.53 × 10^−5^	CASP4, CX3CL1, DAPK1, FOS, FOXO1, ITGA3
SYVN1	transporter	Inhibited	−2	0.000114	ITGA3, ITGA6, ITGB1, ITGB4
ESR2	ligand-dependent nuclear receptor		−1.109	0.000233	CAPN2, FOS, FOXO1, FOXO3, ITGB1, NRG1, SPHK1
GLI1	transcription regulator		1.633	0.000299	FOS, FOXO1, ITGA3, ITGB4, NRG1, SERPINA1
IL6	cytokine		−1.174	0.000412	CASP4, FOS, HSPA5, ITGB1, PPP1R15A, SERPINA1, TNFSF10
P38 MAPK	group		1.071	0.000462	BNIP3, FOS, HSPA5, ITGB4, TNFSF10
TGM2	enzyme	Inhibited	−2	0.000648	BNIP3, DAPK2, ITGA6, ITGB4
STAT1	transcription regulator		1.501	0.000778	CASP4, CX3CL1, FOS, FOXO1, TNFSF10
Growth hormone	group		1.9	0.000822	CX3CL1, FOS, PPP1R15A, TNFSF10
AHR	ligand-dependent nuclear receptor		1.97	0.00109	FOS, HSPA5, ITGA6, PPP1R15A, TP73
Tgf beta	group		−1.154	0.00194	FOS, ITGA6, ITGB1, TNFSF10
IL1	group		1.236	0.0032	FOS, NRG1, SPHK1, TNFSF10
CEBPB	transcription regulator		−1.239	0.00394	DAPK1, FOS, FOXO3, PPP1R15A, SERPINA1
IL13	cytokine		1.199	0.00853	CAPN2, CX3CL1, SERPINA1, SPHK1
CG	complex		1.067	0.00891	CX3CL1, FOS, ITGA3, ITGB1
LEP	growth factor		1.184	0.0105	FOS, FOXO3, HSPA5, TNFSF10
IGF1	growth factor		1.191	0.0199	FOS, FOXO1, HSPA5, ITGA3

## Data Availability

All RNA-seq data were deposited in the Sequence Read Archive (SRA) (BioProject ID: PRJNA807443).

## Supplementary Materials

The following supporting information can be downloaded at: https://www.mdpi.com/article/10.3390/cells11071116/s1, Table S1: Human Autophagy-Related Genes (ARGs) Dataset; Table S2: Primer sequences of qRT-PCR; Table S3: Summary of differentially expressed autophagy-related genes enriched canonical pathways in vitiligo; Table S4: Summary of differentially expressed autophagy-related genes enriched diseases and bio functions in vitiligo; Table S5: Inflammatory response analysis of differentially expressed autophagy-related genes in vitiligo; Table S6: Free radical scavenging analysis of differentially expressed autophagy-related genes in vitiligo; Table S7: Summary of upstream regulator analysis for differentially expressed autophagy-related genes in vitiligo; Table S8: Summary of enriched molecular network analysis for differentially expressed autophagy-related genes in vitiligo. Figure S1: The network of differentially expressed autophagy-related genes involved in the ‘Autophagy’ pathway.
