# Supplementary material for: Identification and Validation of Autophagy-Related Genes in Vitiligo
Source: Cells. 2022 Mar 25;11(7):1116. doi: 10.3390/cells11071116 (PMC8997611; doi:10.3390/cells11071116)
Supplement: Supplementary file 1 [file cells-11-01116-s001.zip › Figure S1.pdf]

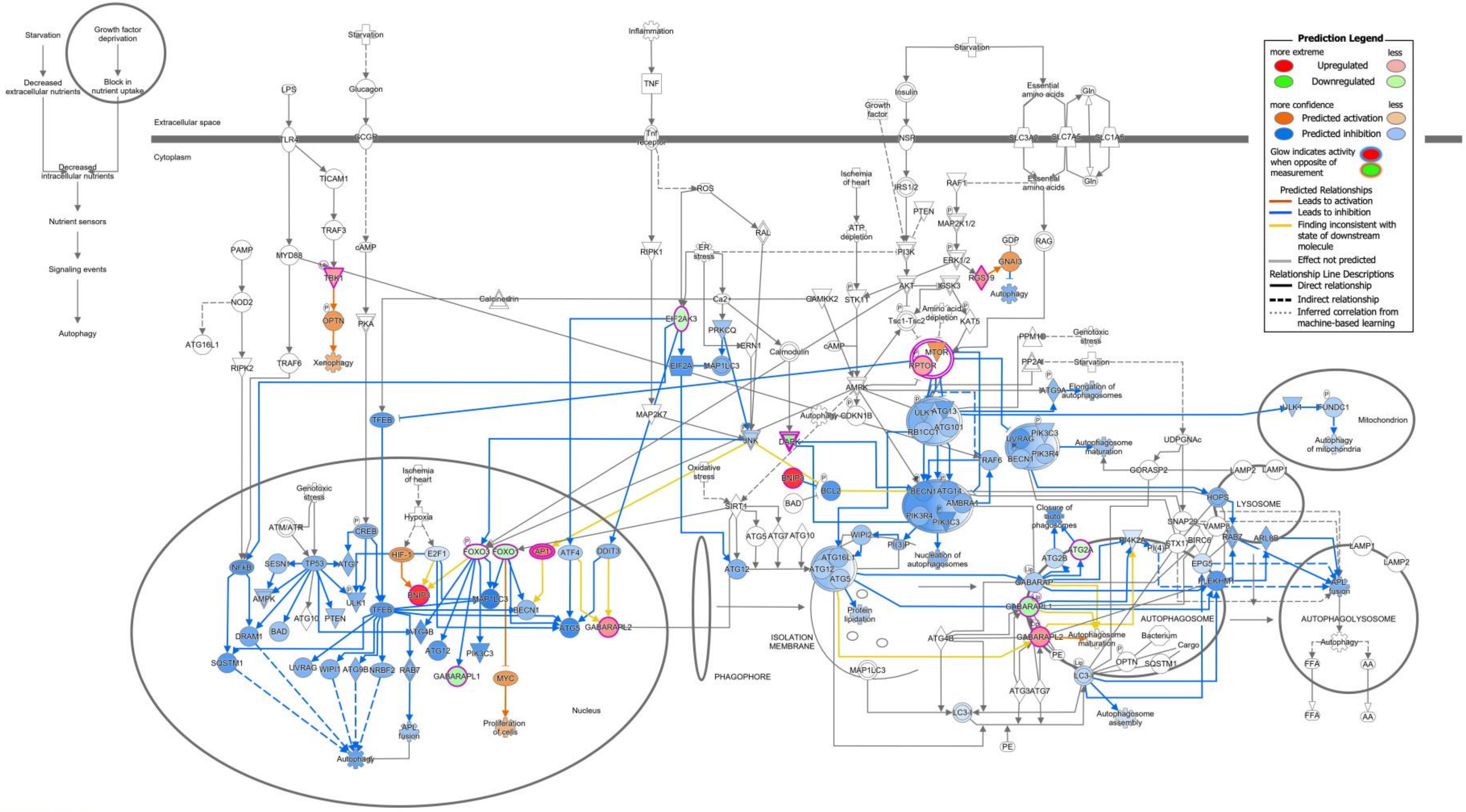

© 2000-2022 QIAGEN. All rights reserved.

Figure S1. The network of differentially expressed autophagy-related genes (DEARGs) involved in the 'Autophagy' pathway. Among 39 DEARGs, 13 genes were considered to be involved in the 'Autophagy' pathway in Ingenuity Pathways Analysis (IPA).
