# Supplementary material for: Identification and Validation of Autophagy-Related Genes in Vitiligo
Source: Cells. 2022 Mar 25;11(7):1116. doi: 10.3390/cells11071116 (PMC8997611; doi:10.3390/cells11071116)
Supplement: Supplementary file 1 [file cells-11-01116-s001.zip › Table S2.pdf]

Table S2: Primer Sequences for qRT-PCR

Gene Names	Upper Primer Sequence (5’-3’)	Lower Primer Sequence (5’-3’)
BNIP3	CATCTCTGCTGCTCTCTCATT	TCATCAAAAGGTGCTGGTG
TNFSF10	TCTCTCTGTGTGGCTGTAACT	AGGGGCTGTTCATACTCTCT
FOS	CGGAGACAGACCAACTAGAAG	GCTGCCAGGATGAACTCTA
PEX3	GCCTTCAAACAAGCTAGAAA	AAAAGAACAACCAGCATACAG
RGS19	CTCCGCATCGTGTAGTTTTGT	TGCAGGATTCTGAGGTCCTAA
DAPK1	GTGTTGCCAGAAGCGATGATA	GGATAATGAGCCCAGAAGGTG
SERPINA1	GCTTACATTTACCCAAACTGTCC	CCATTGCTGAAGACCTTAGTG
GRID1	CTGGATATGCCAGTGCGTGTC	AGTAGGTGATCTTCTCGCTCTGC
CX3CL1	CTGGTGACTTTCCTCTTTGGT	GAGTATGTTGGTGGCTTGATG
